# Narrowband ultraviolet B radiation attenuates nucleus pulposus pyroptosis to ameliorate intervertebral disc degeneration by activating NRF2/KEAP1 antioxidant pathway

**DOI:** 10.3389/fimmu.2025.1663674

**Published:** 2025-10-22

**Authors:** Helou Zhang, Xinyun Tang, Huiqing Zhou, Chengcong Zhou, Zhiguo Zhang, Shuchao Shen, Xuliang Fang, Jiazhe Du, Shenghua Cheng, Jinjie Zhang, Shijia Xing, Zikun Chen, Fangda Fu, Kun Tian, Huan Luo, Jin Pan, Chengliang Wu, Hongfeng Ruan

**Affiliations:** ^1^ Institute of Orthopaedics and Traumatology, The First Affiliated Hospital of Zhejiang Chinese Medical University (Zhejiang Provincial Hospital of Traditional Chinese Medicine), Hangzhou, China; ^2^ The First Clinical Medical College, Zhejiang Chinese Medical University, Hangzhou, China; ^3^ Department of Orthopaedics, The Third Affiliated Hospital of Zhejiang Chinese Medical University, Hangzhou, China; ^4^ Department of Pharmacy, The Second Affiliated Hospital, Zhejiang University School of Medicine, Hangzhou, China; ^5^ School of Architecture, Department of Architecture, China Academy of Art, Hangzhou, China

**Keywords:** intervertebral disc degeneration, narrowband ultraviolet B, NLRP3-mediated pyroptosis, nucleus pulposus, NRF2/KEAP1 antioxidant pathway

## Abstract

Intervertebral disc degeneration (IVDD) is a major cause of low back pain, with nucleus pulposus (NP) cell pyroptosis—a highly inflammatory form of programmed cell death mediated by NLRP3 inflammasome—playing a crucial role in its pathogenesis by triggering inflammatory cascades and accelerating matrix degradation. Although ultraviolet B (UVB) irradiation has shown therapeutic potential in various inflammatory diseases, its effect on IVDD and underlying mechanisms remained unexplored. In this study, we established a lumbar spine instability (LSI)-induced IVDD mouse model and administered UVB irradiation (315 nm, ~1.0944 mW/cm²) for either 2 minutes (UVB+ group, ~0.13 J/cm²) or 4 minutes (UVB++ group, ~0.26 J/cm²) three times weekly over 8 weeks. UVB treatments effectively attenuated IVDD progression, as evidenced by improved behavioral outcomes, preserved disc height, and maintained structural integrity. Both UVB protocols enhanced extracellular matrix homeostasis, reduced cell apoptosis, and suppressed inflammatory responses, with the UVB+ regimen showing superior efficacy. Mechanistically, UVB significantly inhibited NLRP3-mediated pyroptosis by downregulating NLRP3, ASC, CASPASE1, and GSDMD expression through potent activation of the NRF2/KEAP1 antioxidant pathway. Our findings demonstrate that UVB irradiation effectively ameliorates IVDD by activating the NRF2/KEAP1 pathway and subsequently suppressing NLRP3-mediated pyroptosis, with the 2-minute protocol showing optimal therapeutic effects, establishing UVB irradiation as a promising non-invasive therapeutic strategy for IVDD treatment.

## 1 Introduction

Lower back pain (LBP) is one of the most prevalent musculoskeletal disorders worldwide. Its average prevalence rate among adults is approximately 12%, and the chronic lifelong rate is about 40% (1). Intervertebral disc (IVD) degeneration (IVDD) has been identified as a major pathological basis for LBP. Current therapeutic strategies for IVDD primarily consist of conservative treatments or surgical interventions. Conservative approaches often involve pain management regimens, including NSAIDs, muscle relaxants, opioids, and physical therapy, to alleviate symptoms. However, these methods are limited in their ability to reverse disc degeneration, while surgical procedures are associated with considerable risks and complications (2, 3). Therefore, developing effective and minimally invasive therapeutic approaches remains an urgent clinical need in the management of IVDD.

Ultraviolet (UV) light therapy, which emerged as a medical intervention in the early 20^th^ century, encompasses three distinct spectral ranges: UVA (320–400 nm), UVB (280–320 nm), and UVC (100–280 nm) (4). Among these, UVB phototherapy has demonstrated remarkable therapeutic efficacy across various inflammatory and autoimmune conditions, including psoriasis, atopic dermatitis, vitiligo, and systemic lupus erythematosus (5–8). Particularly, narrowband UVB (NB-UVB) therapy has shown significant promise in ameliorating psoriasis symptoms through modulation of systemic inflammation via suppression of multiple inflammatory signaling pathways (9–11). Recent investigations have also revealed UVB’s potent antioxidant effects in conditions such as vitiligo, primarily through activation of endogenous antioxidant defense mechanisms (12). Furthermore, compelling evidence suggests that controlled UVB exposure at a specific wavelength of 316 nm can enhance bone formation, decrease bone resorption, and improve bone mass and mineral density without inducing adverse skin effects (13). These multifaceted therapeutic properties of UVB—particularly its anti-inflammatory and antioxidant effects—suggest its potential application in degenerative conditions beyond dermatological disorders.

The structure of a normal intervertebral disc consists of a central, highly viscous gel-like core, the nucleus pulposus, which is encapsulated by the peripheral annulus fibrosus—a fibrous ring primarily of type II collagen. The nucleus pulposus synthesizes glycosaminoglycans and proteoglycans, molecules critical for the disc's water-retention capacity. The pathophysiology of IVDD is characterized by a chronic degenerative process resulting in progressive structural deterioration, particularly disrupted ECM homeostasis, manifested as reduced AGGRECAN and Collagen 2 (COL2) expression, upregulated matrix-degrading enzymes (ADAMTS5 and MMP3), enhanced inflammatory responses, and accelerated cell apoptosis, ultimately leading to decreased disc height and impaired lumbar mechanical function (14). Recent evidence has highlighted that NP cell pyroptosis, a highly inflammatory form of programmed cell death, plays a crucial role in IVDD pathogenesis, which is primarily mediated by the NLRP3 inflammasome and requires CASPASE1 activation, leading to the release of pro-inflammatory cytokines such as IL-1β and IL-18. The resultant inflammatory microenvironment accelerates ECM homeostasis and further NP cell death, creating a detrimental feedback loop that expedites disc degeneration (15). Consequently, targeting NLRP3-mediated represents a promising therapeutic strategy to prevent IVDD progression.

Clinical evidence supporting UVB’s therapeutic potential in musculoskeletal conditions has emerged from a randomized single-blinded controlled trial involving fifty-two postmenopausal Korean women (older than 65 years) with osteoporosis, which demonstrated that low energy UVB exposure improved bone alkaline phosphatase (ALP) levels and bone formation in vitamin D insufficient group (16), while NB-UVB therapy significantly reduces the expression of inflammatory cytokines in various conditions, particularly IL-18 (17), IL-12, and IL-23 in psoriatic lesions, TNF-α, and IL-6 in PBMCs (18, 19), as well as IL-1β and Soluble interleukin 1 receptor type 1(sIL-1R1) in plasma (20) of patients with psoriasis, thereby suppressing inflammation and improving clinical symptoms. However, whether UVB could modulate NP cell pyroptosis-induced inflammation in the context of IVDD remains obscure.

Oxidative stress has been increasingly recognized as a key trigger for NLRP3-mediated pyroptosis during IVDD progression. The Nuclear factor erythroid 2-related factor 2 (NRF2)/Kelch-like ECH-associated protein 1 (KEAP1) pathway represents a master regulator of cellular antioxidant defense. Under physiological conditions, NRF2 is sequestered by KEAP1 in the cytoplasm. Upon activation, NRF2 translocates to the nucleus and induces the expression of antioxidant genes, including heme oxygenase-1 (HO-1) (21). Numerous studies have demonstrated that activation of the NRF2 pathway can effectively suppress NLRP3 inflammasome assembly and subsequent pyroptotic cell death in various inflammatory disease contexts (22–24). In addition, our latest findings demonstrated that enhancing NRF2-mediated antioxidant response might represent an effective approach to prevent IVDD progression (25, 26). Meanwhile, UVB radiation has demonstrated efficacy in ameliorating the progression of multiple skin disorders by modulating oxidative stress, particularly through targeting this same NRF2 antioxidant pathway (27, 28). Taken together, these studies support the hypothesis that UBV administration exhibits potential to attenuate NP cell pyroptosis and ameliorate the progression of IVDD through activation of the NRF2/KEAP1 antioxidant pathway.

In this study, we investigated the therapeutic effect of UVB against IVDD pathogenesis using a lumbar spine instability (LSI) surgery-induced IVDD mice. Mice underwent 2 or 4 minutes of UVB irradiation (three times per week) for 8 weeks post-surgery. Our findings demonstrate that UVB treatment significantly improved IVDD outcomes by preserving disc structure, maintaining ECM homeostasis, inhibiting apoptosis, and reducing inflammation. Notably, UVB suppressed NP pyroptosis by downregulating NLRP3, ASC, CASPASE1, and GSDMD expression, with mechanistic analysis revealing NRF2/KEAP1 pathway activation as the key regulatory mechanism underlying these therapeutic effects. Our *in vivo* findings provide further insights into the pharmacological properties of UVB concerning IVDD progression, highlighting its potential as a promising therapeutic modality for clinical IVDD management.

## 2 Materials and methods

### 2.1 Animals and experimental design

Male C57BL/6 J mice (4 weeks old, 14–16 g, n = 112) were obtained from the Animal Experimentation Center of Zhejiang Chinese Medical University (Grade SPF, SCXK Shanghai). Animals were housed in a specific pathogen-free (SPF) facility under controlled conditions (12-hour light/dark cycle, temperature 23 ± 2 °C, 55% humidity) with *ad libtum* access to water and standard laboratory chow. Polycarbonate cages without bedding or environmental enrichment SPF standards. All procedures adhered to the ARRIVE guidelines for animal research reporting and were approved by the Ethical Committee of Zhejiang Chinese Medical University (No. 20231030–03).

The mice were randomly divided into four groups (n = 28 per group): Sham (placebo surgery), Model (LSI surgery), UVB+ (LSI surgery + 2-min UVB exposure), and UVB++ (LSI surgery + 4-min UVB exposure). The LSI surgery-induced IVDD model was established as previously described (25). All mice except those in the Sham group, underwent IVDD modeling. Briefly, under isoflurane anesthesia, the L3-L5 spinous processes and associated ligaments were surgically exposed and removed. In the Sham group, only muscle exposure was performed without spinous process removal. Post-surgical care included wound closure and prophylactic gentamicin administration to prevent infection.

NB-UVB treatment commenced three days post-surgery using a fluorescent lamp (314 nm, ~1.0944 mW/cm²) positioned 20 cm above the dorsal region. To ensure consistent UVB delivery, the dorsal fur from the scapula to the sacrum was meticulously trimmed using electric clippers on a weekly basis. This procedure was performed 24 hours prior to the initial UVB irradiation session each week to minimize potential skin irritation and ensure uniform exposure. The UVB+ and UVB++ groups received irradiation three times weekly for 2 minutes (~0.13 J/cm²) and 4 (~0.26 J/cm²) minutes, respectively. Tissue samples (n = 7 per group) were collected at 1, 2, 4, and 8 weeks post-irradiation for subsequent analyses.

### 2.2 Behavioral assessment

Following a 7-day acclimation period, comprehensive behavioral assessments were conducted at 8 weeks post-irradiation by a single investigator under standardized conditions. On testing days, mice were allowed an additional 20-minute habituation period in the testing apparatus before assessment. The following behavioral parameters were evaluated:

#### 2.2.1 Rearing behavior

Spontaneous vertical activity was quantified by recording the frequency of standing on hind limbs (with or without wall contact) over a 5-minute observation period (29).

#### 2.2.2 Mechanical allodynia

Von Frey filament testing was performed using calibrated filaments (0.2-2.0g) applied to the plantar surface of hindpaws. The paw withdrawal threshold was determined as the lowest force eliciting withdrawal responses in at least three out of five trials. Values from both hindpaws were averaged for analysis (29).

#### 2.2.3 Thermal nociception

The hargreaves test using a plantar test apparatus (Ugo Basile, model 55370). The latency to hindpaw withdrawal was measured, with results averaged from ten trials per mouse. A cut-off time of 20 seconds was implemented to prevent tissue damage (30).

#### 2.2.4 Gait analysis

Following one week of pre-training, mice were assessed using the GaitScan system (CleverSys). Animals traversed a confined corridor (50 × 8 cm) with a glass floor under low-light conditions. Three complete runs were recorded per mouse and analyzed using TreadScan™ 2.0 software (29).

### 2.3 Micro-CT analysis

Lumbar vertebrae were scanned using a high-resolution micro-CT tomography system (Skyscan 1176, Bruker Micro-CT N.V., Kontich, Belgium) at a 90 kV/300 μA current with 9 μm resolution. Three-dimensional reconstructions were generated using CTVol v2.2 software. Lumbar (L) 3-L4 IVD height was calculated by averaging anterior, middle, and posterior measurements from coronal images.

### 2.4 Histological, and immunological analysis

Tissue specimens underwent sequential processing, including fixation (4% paraformaldehyde, 72 h), decalcification (14% EDTA, pH 7.4, 3 weeks), and paraffin embedding. Five-µm coronal sections were prepared for histological and immunological analyses. H&E staining was performed for general morphological assessment, with NP scores evaluated blindly according to Norcross criteria (31). For immunohistochemistry (IHC), sections were incubated overnight at 4°C with primary antibodies against matrix proteins (COL2, AGGRECAN, 1:300), degradative enzymes (ADAMTS5, MMP3, 1:300), apoptotic markers (CASPASE3, BAX, BCL2, 1:500), inflammatory factors (IL-6, TNF-α, IL-1β, IL-18, 1:500), and pyroptosis-related proteins (NLRP3, ASC, CASPASE1, GSDMD, 1:500). Detection was achieved using a horseradish peroxidase-conjugated streptavidin detection system (ZSGB-BIO, China) with hematoxylin counterstaining (Sigma-Aldrich, USA). For immunofluorescence (IF) analysis, sections were incubated with fluorescent-conjugated secondary antibodies for 1 hour at room temperature. Quantitative analysis was performed using Image-Pro Plus software (Version 6.0) by observers blinded to the experimental groups, with all experiments conducted in triplicate.

### 2.5 TUNEL assay

Apoptotic cells in IVD tissues were examined using terminal deoxynucleotidyl transferase dUTP nick end labeling (TUNEL) by the TUNEL Bright Green Apoptosis Detection Kit (Vazyme Biotech, Nanjing, China) according to the manufacturer’s protocol. TUNEL-positive cells were quantified by examining three randomly selected fields from three independent sections per sample. The apoptotic rate was calculated as the ratio of TUNEL-positive cells to total DAPI-stained nuclei. All analyses were performed by investigators blinded to the experimental groups.

### 2.6 Statistical analysis

Data are presented as means ± standard deviation (SD). Statistical analyses were performed using GraphPad Prism 9.0 software. Normal distribution and variance homogeneity were assessed prior to applying one-way ANOVA with least significant difference (LSD) *post-hoc* testing for multiple comparisons. Statistical significance was defined as *p* < 0.05.

## 3 Results

### 3.1 UVB irradiation ameliorates behavioral function and maintains disc height in LSI surgery-induced IVDD mice

**Figure 1:**
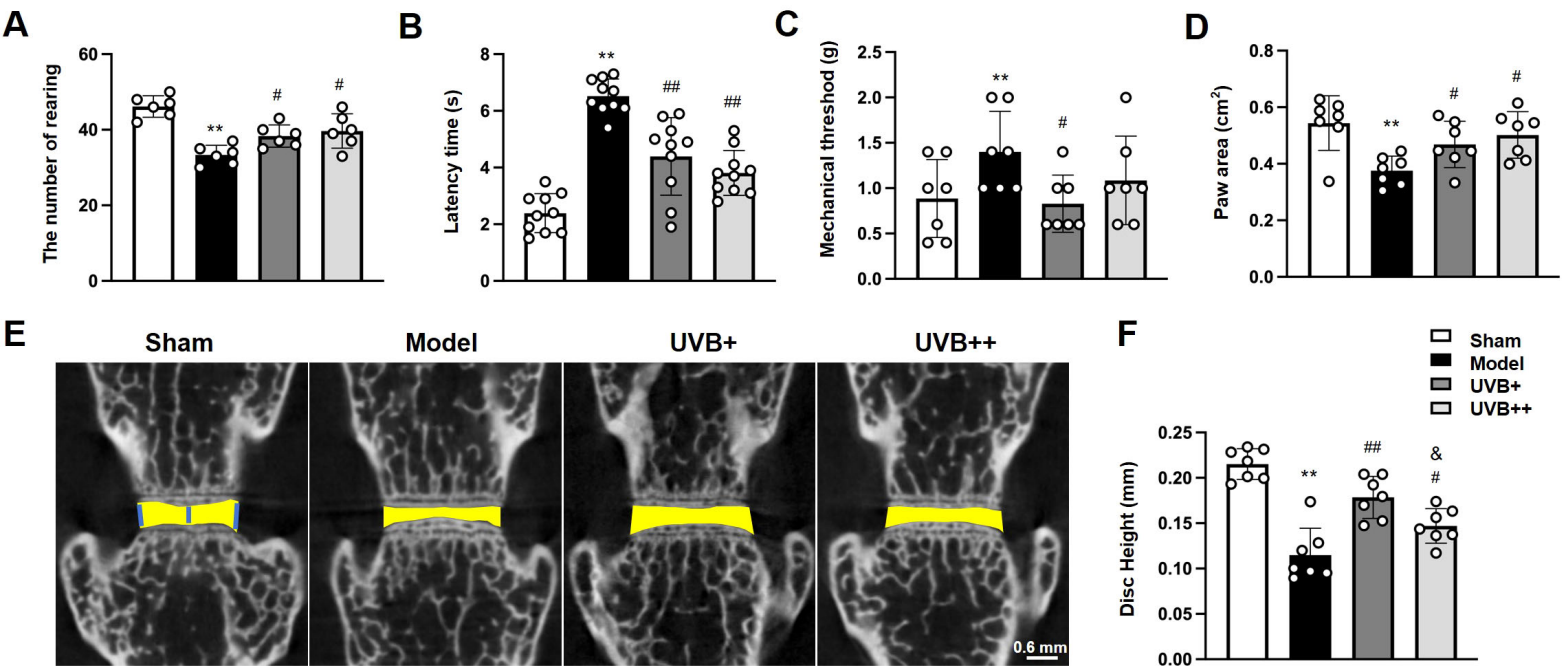
Middle-wavelength UVB irradiation ameliorates behavioral deficits and preserves disc height in LSI-induced IVDD mice. **(A-D)** Behavioral assessments after 8-week UVB treatment, including **(A)** rearing frequency test for spontaneous activity, **(B)** thermal nociceptive response in Hargreaves test, **(C)** mechanical pain threshold in Von-Frey test, and **(D)** paw area measurement in gait analysis. **(E)** Representative μCT images showing disc height changes in L4-L5 segments. **(F)** Quantitative analysis of disc height index. Data are presented as mean ± SD. ^**^ *p* < 0.01 *vs* Sham group; ^#^ *p* < 0.05, ^##^ *p* < 0.01 *vs* Model group; ^&^ *p* < 0.05 *vs* UVB+ group. n = 6 per group.

To evaluate the therapeutic potential of UVB on IVDD development, mice underwent LSI surgery followed by UVB irradiation (wavelength: 314 nm, power density: 1.0944 mW/cm²) for either 2 minutes or 4 minutes triweekly over 8 weeks. Comprehensive behavioral assessments revealed that IVDD mice exhibited significant motor impairments, evidenced by a 28% reduction in rearing frequency compared to Sham controls (33.33 ± 2.36 *vs.* 46.17 ± 2.61, *p* < 0.01). Both UVB treatments effectively improved rearing behavior, with frequencies restored to 38.33 ± 2.69 (UVB+) and 39.67 ± 4.15 (UVB++) (**Figure 1A**). Thermal nociception testing showed significantly increased latency time in IVDD mice (6.52 ± 0.57s *vs.* 2.38 ± 0.66s in Sham, *p* < 0.01), which was normalized by both UVB treatments (4.49 ± 1.30s in UVB+ and 3.91 ± 0.76s in UVB++) (**Figure 1B**). Similarly, mechanical pain threshold was elevated in IVDD mice (1.31 ± 0.42g *vs.* 0.89 ± 0.35g in Sham, *p* < 0.01) and normalized by UVB treatment (0.89 ± 0.30g in UVB+ and 1.17 ± 0.46g in UVB++) (**Figure 1C**). Gait analysis revealed reduced paw area in IVDD mice (0.38 ± 0.05 cm^2^ *vs.* 0.54 ± 0.09 cm^2^ in Sham, *p* < 0.01), which was significantly improved by both UVB treatments (0.54 ± 0.10 cm^2^ in UVB+ and 0.50 ± 0.08 cm^2^ in UVB++) (**Figure 1D**).

Micro-CT analysis of the L4-L5 IVD revealed significant disc height reduction in IVDD mice (0.11 ± 0.01 mm *vs.* 0.22 ± 0.04 mm in Sham, representing a 48% decrease, *p* < 0.01). Both UVB treatments effectively preserved disc height, with UVB+ showing superior efficacy (0.18 ± 0.02 mm) compared to UVB++ (0.15 ± 0.02 mm) (**Figures 1E, F**).

### 3.2 UVB irradiation preserves structural integrity and ECM homeostasis in IVDD mice

**Figure 2:**
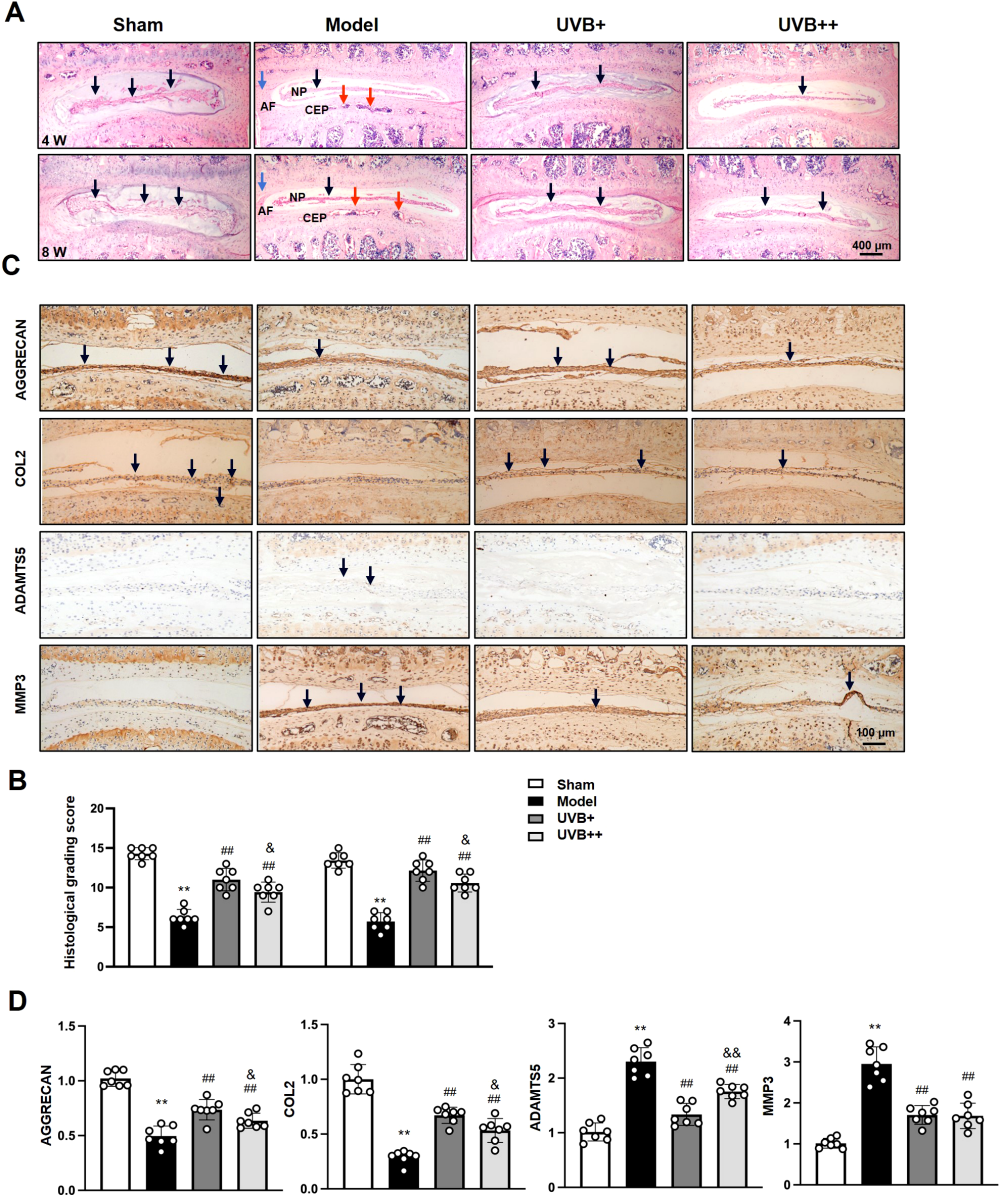
UVB treatment preserves disc structure and ECM homeostasis. **(A)** Representative H&E staining of L4-L5 IVDs at 4 and 8 weeks post-surgery. Black arrows indicate NP area, red arrows indicate CEP area, and blue arrows indicate AF area. Scale bar = 400 μm. **(B)** Histological grading scores. **(C)** Representative IHC images showing ECM-related protein expression. Scale bar = 100 μm. **(D)** Quantification of AGGRECAN, COL2, ADAMTS5, and MMP3 expression levels. Data are presented as mean ± SD. ^**^ *p* < 0.01 *vs* Sham group; ^##^ *p* < 0.01 *vs* Model group; ^&^ *p* < 0.05 *vs* UVB+ group. n = 6 per group.

To investigate the effects of UVB irradiation on IVD structure and ECM homeostasis, histopathological analysis of L4-L5 IVD revealed progressive degenerative changes in IVDD mice, with HE staining showing significant NP height reduction at week 4, which further deteriorated by week 8. The Model group also exhibited distinct morphological changes, including disorganized annulus fibrosus (AF) structure and notable cartilaginous endplate (CEP) calcification. Both UVB treatments effectively preserved disc height and tissue architecture, with maintained NP structure and reduced CEP ossification (**Figure 2A**). Quantitative histological scoring confirmed these observations, showing that the Model group scores decreased significantly (5.71 ± 1.11 *vs.* 13.43 ± 0.98 in Sham, *p* < 0.01). While both UVB treatments maintained significantly higher scores, UVB+ demonstrated superior protective effects (12.14 ± 1.34) compared to UVB++ (10.57 ± 1.13, *p* < 0.05 between UVB groups) (**Figure 2B**).

To assess the effect of UVB irradiation on ECM metabolism of IVDs, we determined the expression levels of major ECM components, AGGRECAN and COL2, and their corresponding degradases ADAMTS5 and MMP3, using IHC analysis. Substantial alterations in matrix homeostasis were observed following LSI surgery. AGGRECAN expression decreased by 50% (0.50 ± 0.10 *vs.* 1.01 ± 0.08 in Sham, *p* < 0.01), and COL2 levels reduced by 70% (0.30 ± 0.06 *vs.* 1.00 ± 0.14 in Sham, *p* < 0.01) in the Model group. Notably, while both UVB treatments improved ECM homeostasis, UVB+ exhibited superior efficacy in preserving AGGRECAN (0.74 ± 0.10 *vs.* 0.63 ± 0.07 in UVB++, *p* < 0.05) and COL2 levels (0.67 ± 0.07 *vs.* 0.52 ± 0.10 in UVB++, *p* < 0.05), as well as suppressing ADAMTS5 expression (1.32 ± 0.23 *vs.* 1.74 ± 0.14 in UVB++, *p* < 0.05). Both treatments demonstrated comparable inhibitory effects on MMP3 expression (1.70 ± 0.23 *vs.* 1.65 ± 0.31 in UVB+, *p* > 0.05) (**Figures 2C, D**).

### 3.3 UVB irradiation reduces NP cell apoptosis in IVDD mice

**Figure 3:**
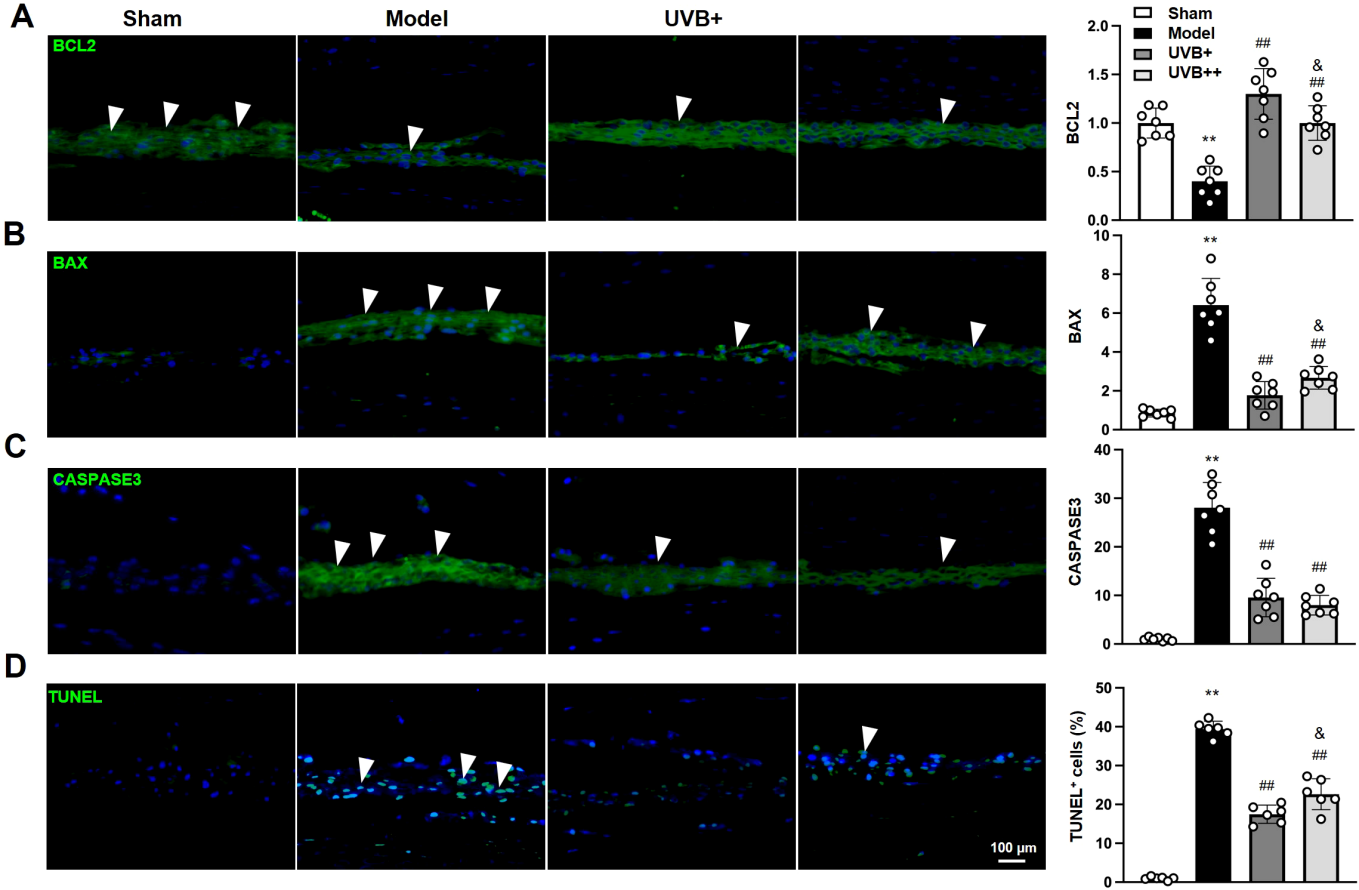
UVB irradiation suppresses NP cell apoptosis in IVDD mice. **(A-D)** Representative immunofluorescence images and quantification of **(A)** BCL2, **(B)** BAX, **(C)** Cleaved-CASPASE3, and **(D)** TUNEL-positive cells in NP tissues. Data are presented as mean ± SD. ^**^ *p* < 0.01 *vs* Sham group; ^##^ *p* < 0.01 *vs* Model group; ^&^ *p* < 0.05 *vs* UVB+ group. n = 6 per group.

To identify the effect of UVB irradiation on the apoptosis of IVD cells, the expression of key apoptotic-related markers, BCL2, BAX, CASPASE3 were determined using IF analysis. The results showed significant dysregulation of apoptotic markers in IVDD mice. The model group showed a 60% decrease in anti-apoptotic BCL2 expression and 7.5-fold and 28.1-fold increases in pro-apoptotic markers including BAX and CASPASE3 in NP tissues, compared to the Sham mice. Notably, UVB+ demonstrated superior anti-apoptotic effects compared to UVB++, with higher BCL2 expression (1.29 ± 0.25 *vs.* 0.98 ± 0.17, *p* < 0.05) and lower BAX levels (1.69 ± 0.73 *vs.* 2.60 ± 0.58, *p* < 0.05), while both treatments showed comparable reduction in CASPASE3 expression (UVB+: 9.54 ± 3.93, UVB++: 8.01 ± 2.03) (**Figures 3A-C**). Similarly, the anti-apoptosis effect of UVB irradiation was further confirmed by TUNEL staining, showing significantly elevated apoptotic cells in the Model group (39.30 ± 1.94% *vs.* 0.96 ± 0.47% in Sham, *p* < 0.01), with UVB+ showing more potent anti-apoptotic effects (17.46 ± 2.36%) compared to UVB++ (22.24 ± 3.67%, *p* < 0.05) (**Figure 3D**).

### 3.4 UVB irradiation inhibits inflammatory response in IVDD mice

**Figure 4:**
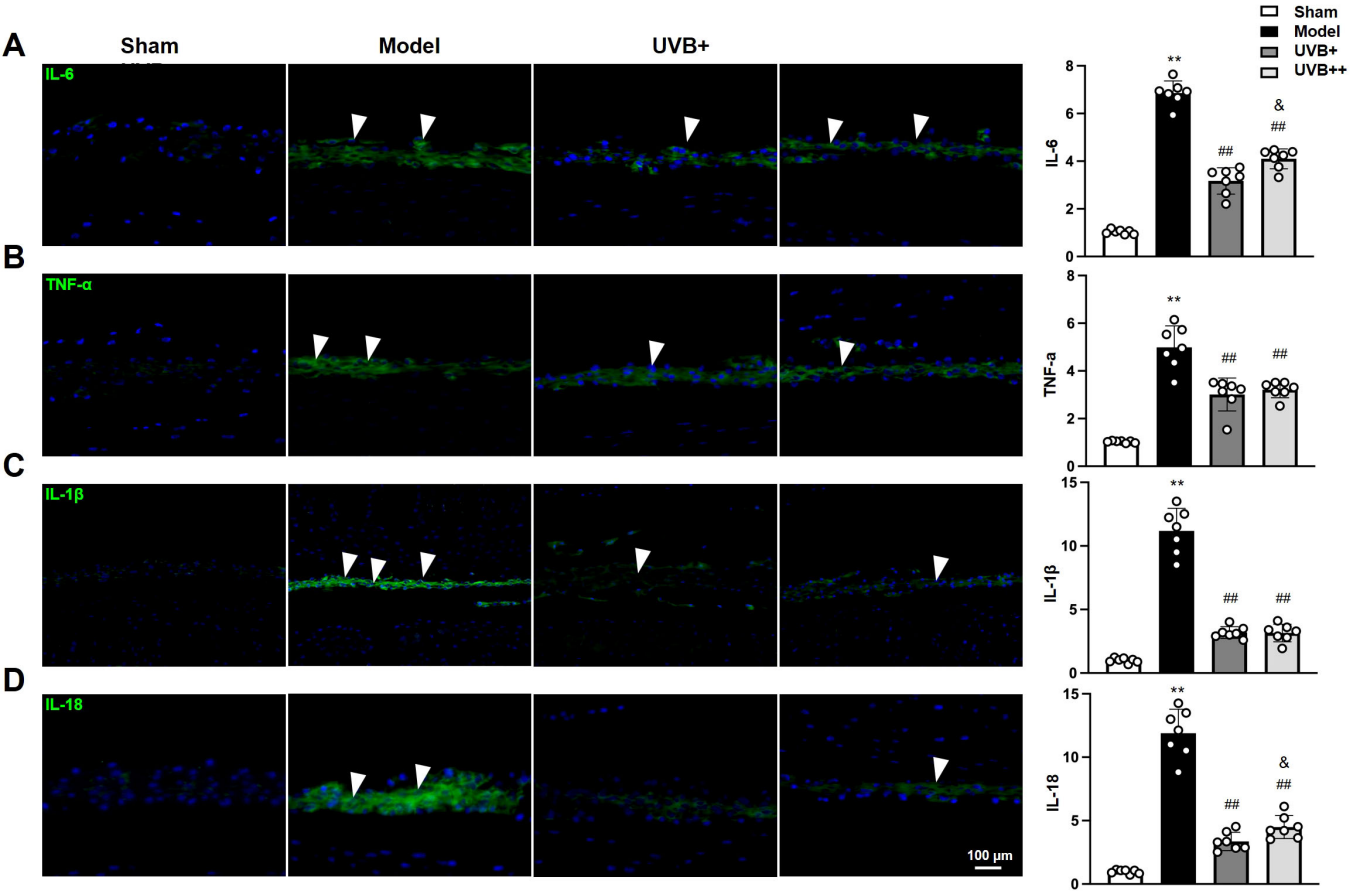
UVB treatment attenuates inflammatory responses in degenerative IVDs. **(A-D)** Representative immunofluorescence images and quantification of pro-inflammatory cytokines **(A)** IL-6, **(B)** TNF-α, **(C)** IL-1β, and **(D)** IL-18 expression in NP tissues. Data are presented as mean ± SD. ^**^ *p* < 0.01 *vs* Sham group; ^##^ *p* < 0.01 *vs* Model group; ^&^ *p* < 0.05 *vs* UVB+ group. n = 6 per group.

Given the critical role of inflammation in IVDD pathogenesis, we next investigated whether UVB irradiation modulates inflammatory responses within the degenerative IVDs, the expressions of inflammatory mediators (IL-1β, IL-18, IL-6, and TNF-α) were examined using IF assay. As expected, LSI surgery significantly induced the upregulation of these inflammatory mediators in the NP tissues of IVDD mice, with the most pronounced increases observed in pyroptosis-associated cytokines IL-1β (11.13-fold) and IL-18 (12.2-fold) compared to Sham controls (*p* < 0.01). Other inflammatory markers also showed significant elevation, with IL-6 and TNF-α increasing by 6.7-fold and 4.8-fold, respectively (*p* < 0.01). Notably, both UVB treatments effectively suppressed these inflammatory markers, with UVB+ demonstrating superior anti-inflammatory effects compared to UVB++ in reducing IL-18 (UVB+: 3.39 ± 0.69, UVB++: 4.50 ± 0.83) and IL-6 (3.11 ± 0.52 *vs.* 4.11 ± 0.39, *p* < 0.05) levels. Meanwhile, both treatments showed comparable efficacy in suppressing IL-1β (UVB+: 3.17 ± 0.38, UVB++: 3.13 ± 0.61) and TNF-α (3.00 ± 0.62 *vs.* 3.27 ± 0.32, *p* < 0.05) expression (**Figures 4A-D**).

### 3.5 UVB irradiation suppresses NLRP3-mediated pyroptosis in IVDD mice

**Figure 5:**
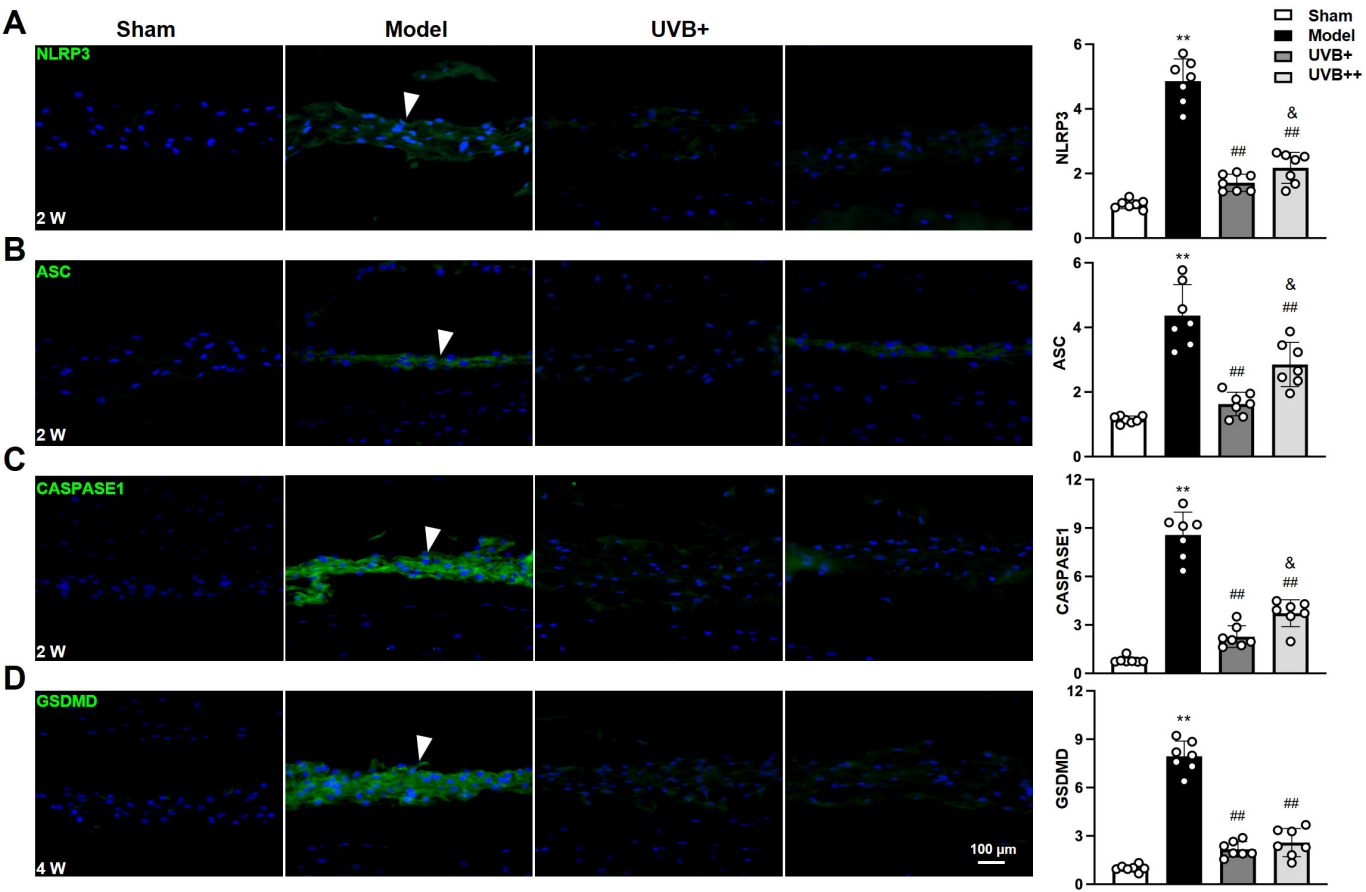
UVB exposure inhibits NLRP3-mediated pyroptosis in NP cells. **(A-D)** Representative immunofluorescence images and quantification of pyroptosis-related proteins **(A)** NLRP3, **(B)** ASC, **(C)** CASPASE1, and **(D)** GSDMD at indicated time points. Data are presented as mean ± SD. ^**^ *p* < 0.01 *vs* Sham group; ^##^ *p* < 0.01 *vs* Model group; ^&^ *p* < 0.05 *vs* UVB+ group. n = 6 per group.

Given the marked upregulation of pyroptosis-associated cytokines IL-1β and IL-18 in IVDD progression and their marked suppression following UVB irradiation treatment, we further investigated the role of NLRP3 inflammation pathway in UVB-mediated protection against IVDD by examining the expression of key pyroptosis-related proteins, including NLRP3, ASC, CASPASE-1, and GSDMD. IF analysis revealed significant activation of the pyroptotic cascade in the NP tissues of IVDD mice, characterized by significant increases in NLRP3 (4.7 ± 0.68-fold), ASC (3.8 ± 0.90-fold), CASPASE1 (10.3 ± 1.34-fold), and GSDMD (8.0 ± 0.7-fold) compared to Sham controls (all *p* < 0.01). Notably, UVB+ demonstrated superior efficacy in suppressing all pyroptotic markers compared to UVB++, as evidenced by lower expression levels of NLRP3 (1.71 ± 0.27 *vs.* 2.16 ± 0.47, *p* < 0.05), ASC (1.62 ± 0.36 *vs.* 2.78 ± 0.63, *p* < 0.05), and CASPASE-1 (2.29 ± 0.63 *vs.* 3.60 ± 0.80, *p* < 0.05). Although both treatments showed similar effectiveness in reducing GSDMD expression (UVB+: 2.15 ± 0.45, UVB++: 2.49 ± 0.86) (**Figures 5A-D**), the overall results indicate that UVB irradiation effectively inhibits NLRP3-mediated pyroptosis in IVDD mice, with UVB+ showing more potent suppressive effects.

### 3.6 UVB irradiation activates the NRF2/KEAP1 antioxidant pathway in IVDD mice

**Figure 6:**
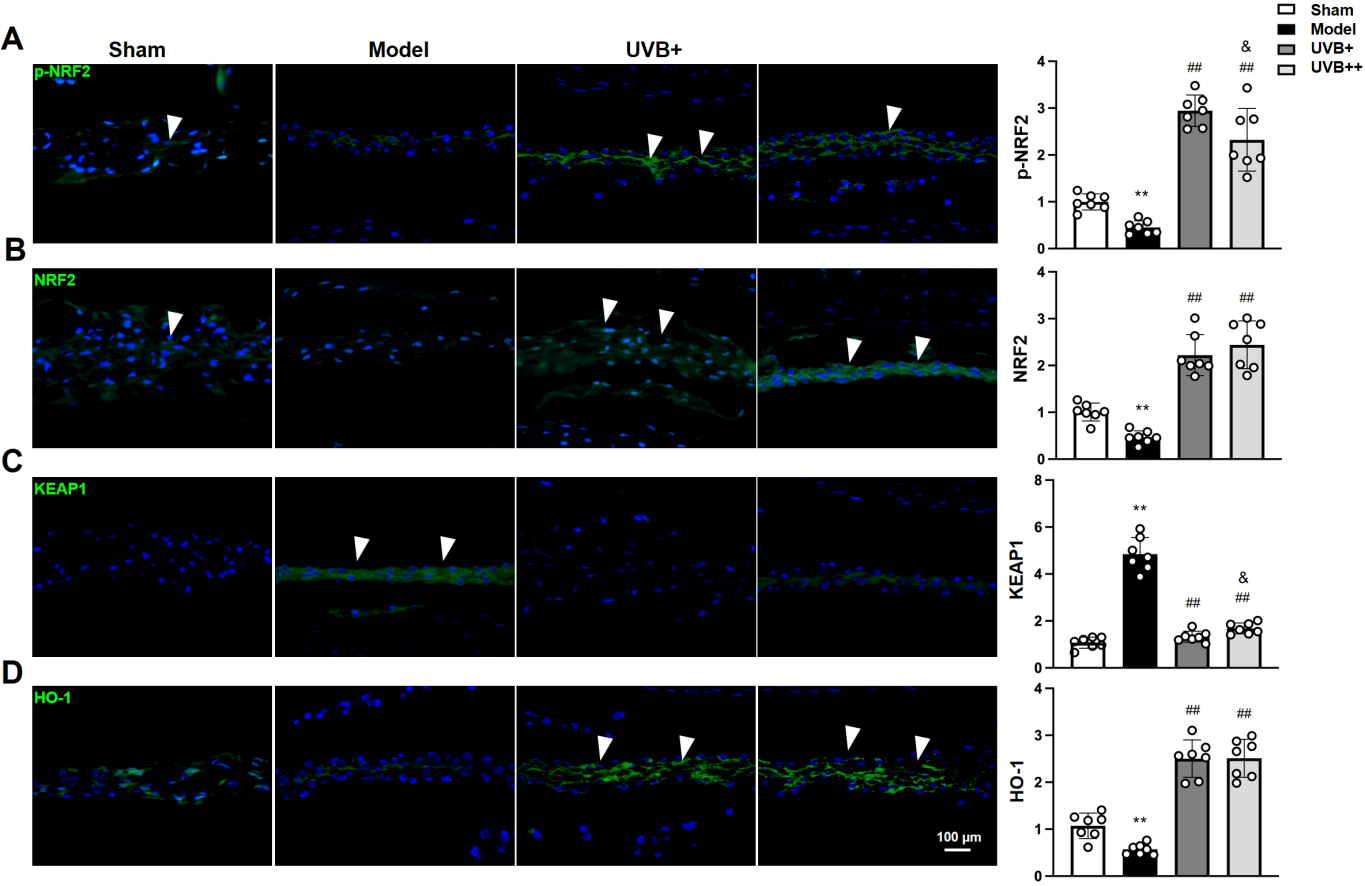
UVB activates NRF2/KEAP1 antioxidant pathway in degenerative discs. **(A-D)** Representative immunofluorescence images and quantification of **(A)** phosphorylated NRF2, **(C)** total NRF2, **(E)** KEAP1, and **(G)** HO-1 expression in NP tissues. Data are presented as mean ± SD. ^**^ *p* < 0.01 *vs* Sham group; ^##^ *p* < 0.01 *vs* Model group; &*p* < 0.05 *vs* UVB+ group. n = 6 per group.

To further elucidate the mechanisms underlying UVB’s anti-pyroptotic effects, we examined the NRF2/KEAP1 antioxidant pathway by analyzing the expression and phosphorylation levels of the antioxidant response protein NRF2, along with its negative regulator KEAP1 and downstream target HO-1. The results revealed significant suppression of this pathway in IVDD mice, manifested by marked reductions in p-NRF2 (0.44 ± 0.13 *vs.* 0.99 ± 0.16 in Sham, *p* < 0.01), total NRF2 (0.46 ± 0.21 *vs.* 0.99 ± 0.19 in Sham, *p* < 0.01), and HO-1 (0.56 ± 0.11 *vs.* 1.06 ± 0.23 in Sham, *p* < 0.01), accompanied by elevated KEAP1 expression (4.83 ± 0.72 *vs.* 1.03 ± 0.22 in Sham, *p* < 0.01). Both UVB treatments effectively restored pathway activity, with UVB+ showing superior effects in enhancing p-NRF2 expression (2.91 ± 0.33 *vs.* 2.29 ± 0.61 in UVB++, *p* < 0.05) and suppressing KEAP1 levels (1.31 ± 0.23 *vs.* 1.69 ± 0.22 in UVB++, *p* < 0.05). Interestingly, both treatments demonstrated comparable efficacy in upregulating total NRF2 (UVB+: 2.25 ± 0.41, UVB++: 2.45 ± 0.49) and HO-1 (UVB+: 2.49 ± 0.37, UVB++: 2.52 ± 0.37) (**Figures 6A-D**). These findings suggest that UVB irradiation protects against IVDD progression by activating the NRF2/KEAP1 antioxidant pathway, with UVB+ showing more potent effects on key regulatory components.

## 4 Discussion

**Figure 7:**
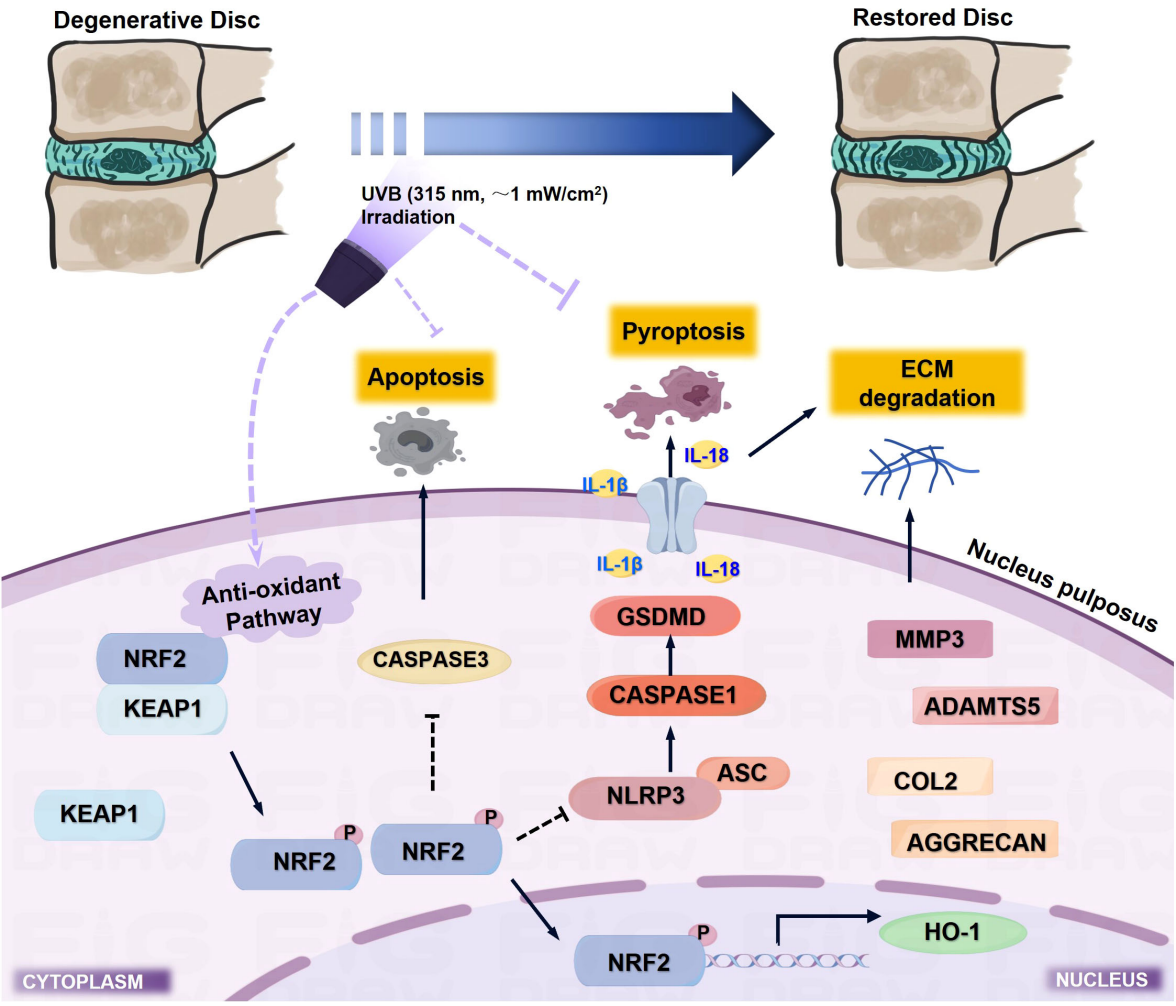
Schematic illustration of the molecular mechanism underlying UVB-mediated protection against IVDD progression. UVB irradiation (315 nm, ~1 mW/cm²) activates the NRF2/KEAP1 antioxidant pathway, which suppresses NLRP3-mediated pyroptosis and subsequent inflammatory cascade, while inhibiting apoptosis and ECM degradation, ultimately leading to preserved disc structure and function.

IVDD is characterized by progressive structural deterioration and functional impairment, significantly impacting patients’ quality of life. Despite advances in understanding its pathogenesis, current treatments remain largely symptomatic, highlighting the urgent need for effective therapeutic strategies (32). The complex interplay between oxidative stress and inflammation in IVDD progression suggests that interventions targeting these pathways might offer promising therapeutic potential. UVB irradiation has shown remarkable therapeutic efficacy in various inflammatory conditions through its immunomodulatory and antioxidant properties (11, 33, 34); however, whether UVB could ameliorate IVDD and its underlying mechanisms remained unexplored. In the present study, we demonstrated that UVB irradiation effectively attenuated LSI surgery-induced IVDD progression, as evidenced by improved behavioral outcomes, preserved disc height, and maintained ECM homeostasis. Notably, the 2-minute UVB treatment showed superior therapeutic effects compared to the 4-minute regimen, particularly in preserving disc structure and ECM composition. Moreover, we identified that UVB treatment significantly suppressed NLRP3-mediated pyroptosis in NP cells, manifested by reduced expression of pyroptotic markers and associated inflammatory cytokines. Mechanistically, we uncovered that UVB treatment suppressed NLRP3-mediated pyroptosis in NP cells by downregulating key pyroptosis-related proteins, including NLRP3, ASC, GSDMD, and CASPASE1, likely through enhanced activation of NRF2/KEAP1 antioxidant pathway (**Figures 7**). These findings not only establish UVB irradiation as a promising therapeutic strategy for IVDD but also reveal novel regulatory mechanism underlying UVB-mediated disc protection.

UVB phototherapy has been widely used in clinical practice for decades, particularly in treating inflammatory skin conditions such as psoriasis and atopic dermatitis, with well-established safety profiles and standardized protocols. For instance, in a case of generalized lichen nitidus, a twice-weekly regimen of 0.25 and 0.15 J/cm² NB-UVB irradiation over 8 to 10 weeks, resulting in cumulative doses of 5.36 and 3.05 J/cm², led to the complete clearance of the majority of lesions (35). Similarly, in a study involving five patients with moderate to severe atopic dermatitis, a total dose of 9.2 J/cm² delivered over 19 sessions significantly alleviated symptoms within three weeks (36). Additionally, ten patients with stable plaque psoriasis treated with NB-UVB at a cumulative dose of 9.8 J/cm² over 14 sessions showed a notable reduction in psoriasis area and severity index scores (37). In our study, the UVB emitter with an output of 1.09 mW/cm² delivered radiation doses of 0.13 J/cm² and 0.26 J/cm² for 2-min and 4-min exposures, respectively. Over an 8-week treatment period, this protocol resulted in cumulative doses of 3.12 J/cm² and 6.24 J/cm², demonstrating consistency with the therapeutic parameters reported in these clinical studies. Our findings extend UVB’s therapeutic potential to IVDD treatment, offering several unique advantages. First, UVB irradiation represents a non-invasive intervention that can be repeatedly administered without accumulative tissue damage. Second, the differential effects observed between UVB+ and UVB++ treatments suggest a critical therapeutic window, where the 2-minute protocol demonstrates optimal efficacy in preserving disc height and ECM homeostasis while potentially minimizing side effects. Moreover, the accessibility and cost-effectiveness of UVB therapy make it particularly attractive for long-term IVDD management. Although our current study utilized substantially lower UVB doses with shorter exposure durations, we recognize the potential risk of cutaneous damage in future studies involving chronic or escalated-dose protocols. To mitigate this risk, subsequent experiments will incorporate a combined approach of localized UVB irradiation with topical antioxidant administration as a protective measure against UV-induced genotoxicity.

The maintenance of ECM homeostasis is crucial for preserving disc function (15). Our results demonstrate that UVB treatment effectively prevents ECM degradation by simultaneously promoting matrix synthesis and inhibiting catabolic processes. The superior efficacy of UVB+ in maintaining AGGRECAN and COL2 levels while suppressing ADAMTS5 suggests a more balanced regulation of ECM metabolism compared to UVB ++. This optimal matrix homeostasis directly contributes to preserved disc height and improved behavioral outcomes, establishing a clear structure-function relationship in UVB-mediated IVDD protection.

Extensive studies have demonstrated that both apoptosis and pyroptosis contribute to IVDD progression, with pyroptosis uniquely capable of triggering inflammatory cytokine bursts while apoptosis does not (38, 39). Emerging evidence suggests UVB therapy can target and inhibit cell apoptosis while simultaneously suppressing the secretion of inflammatory factors including IL-18, IL-6, TNF-α, and IL-1R, thereby demonstrating therapeutic efficacy in the management of inflammatory dermatological conditions such as atopic dermatitis and psoriasis (17, 18, 34, 40), suggesting its potential therapeutic effects through modulation of both apoptotic and pyroptotic pathways. Indeed, our findings demonstrate that UVB effectively suppressed both cell death pathways in IVDD, as evidenced by restored BCL2/BAX balance and reduced CASPASE3 expression, along with significant inhibition of the pyroptotic cascade. The dramatic upregulation of IL-1β and IL-18 in the Model group, coupled with elevated NLRP3 inflammasome components, indicates the prominent role of pyroptosis in IVDD pathogenesis. UVB treatment, particularly the UVB+ protocol, effectively suppressed both the pyroptotic cascade and apoptotic pathway, with TUNEL staining confirming the comprehensive anti-cell death effects. This dual regulation supports our previous findings (25) that pyroptosis functions upstream of apoptosis in IVDD progression, where UVB primarily targets early pyroptotic pathways through NRF2/KEAP1 activation, subsequently reducing inflammatory cytokine-mediated secondary apoptosis, distinguishing it from conventional anti-inflammatory treatments.

The NRF2/KEAP1 signaling pathway serves as a master regulator of cellular antioxidant responses in IVDD progression by orchestrating the transcription of numerous cytoprotective genes, maintaining redox homeostasis, and suppressing inflammatory responses (41). Studies have demonstrated that extracellular nanovesicles, slowed the progression of IVDD by inhibiting nucleus pulposus cell ferroptosis via the NRF2/KEAP1 signaling pathway, and even by promoting m6A demethylation of Nrf2 (42, 43). Emerging evidence suggests that both pharmacological and physical interventions targeting NRF2 signaling exhibit therapeutic potential. For instance, chemical compounds such as senolytic agent quercetin (44), Verapamil (45) have been shown to enhance NRF2-mediated cytoprotective functions, thereby mitigating IVDD progression. Similarly, physical modalities have also shown significant therapeutic effects through NRF2 activation. Moderate mechanical stress has been found to alleviate osteoarthritis progression through NRF2 activation (46), and low-intensity pulsed ultrasound has demonstrated the ability to maintain alveolar bone homeostasis by modulating NRF2 signaling in periodontitis models (47). Moreover, inhibition of oxidative stress has been identified as a key mechanism in UVB therapy for various skin disorders (48–50), particularly through activation of the NRF2/KEAP1 antioxidant pathway (27, 28). The most intriguing finding of our study is the significant activation of the NRF2/KEAP1 pathway by UVB treatment. Notably, UVB not only restored but enhanced NRF2 pathway activity beyond physiological levels, while normalizing elevated KEAP1 expression. This unique activation pattern suggests that UVB might provide superior antioxidant protection compared to conventional NRF2 activators. The stronger activation of this pathway by UVB+ compared to UVB++ correlates well with its superior therapeutic effects, indicating that appropriate UVB dosing is crucial for optimal pathway modulation. However, the causal relationship between the NRF2/KEAP1 pathway and pyroptosis observed in our study requires further validation. Future investigations employing NRF2-specific inhibitors or genetic knockout models would be essential to establish a definitive mechanistic link.

Current evidence suggests that phototherapeutic modalities can directly target the human musculoskeletal system to achieve therapeutic effects, indicating the presence of photosensitive receptors capable of receiving and transmitting light signals in musculoskeletal tissues (51, 52). Although direct evidence of UVB acting on bone remains limited, studies have demonstrated that 408 nm visible light can penetrate up to 1 mm into the skin (53). In our experimental model, the murine epidermal thickness is approximately 0.4 mm, with vertebral bodies and IVDs located directly beneath the skin. Given that UVB wavelengths range between 290–320 nm, we selected NB-UVB with a peak emission at 315 nm, which is close to the most widely reported therapeutic wavelength (311 ± 2 nm). This choice was based on its enhanced penetration depth, improved energy concentration, and superior safety-profile compared to broadband UVB (6). However, whether UVB directly penetrates the epidermis to act on IVDs or exerts its effects indirectly through vitamin D-mediated pathways requires further experimental validation.

Several limitations of our study warrant further investigation. While the LSI surgery-induced IVDD model recapitulates key features of human disc degeneration, the acute nature of this model might not fully represent the chronic progression of human IVDD. Although we demonstrated that UVB irradiation significantly improved the local antioxidant status of IVDs, the precise regulatory mechanisms remain to be elucidated. UVB therapy has been shown to significantly affect serum 25-hydroxyvitamin D and total vitamin D levels (54), which has been recognized as crucial modulators of systemic antioxidant status (55), particularly through the NRF2/KEAP1 pathway (56), inflammatory responses (57, 58), and cellular pyroptosis (59–61). Recent studies have demonstrated that vitamin D receptor (VDR) activation can upregulate the NRF2/HO-1 signaling pathway to suppress ferroptosis and stimulate Nrf2/GPX4 signaling pathways (62, 63). Moreover, clinical studies on VDR polymorphisms (64) and experimental evidence from animal models (65, 66) have consistently demonstrated the protective role of vitamin D in IVDD through suppressing inflammatory responses (67, 68). Indeed, our preliminary data demonstrated that UVB treatment effectively restored the abnormally decreased serum 1,25-(OH)_2_-Vitamin D_3_ levels in ovariectomized-induced osteoporotic mice (data not shown), suggesting a potential systemic mechanism requiring further investigation. The therapeutic efficacy observed in our study may result from synergistic effects between direct NRF2/KEAP1 activation and vitamin D-mediated systemic responses, as recent evidence suggests potential mechanistic crosstalk between VDR signaling and NRF2 pathways that warrants further investigation. Although no adverse effects were observed in the current experimental setting, comprehensive safety assessments including detailed evaluation of skin integrity, DNA damage markers, long-term carcinogenic risks, bone integrity, surrounding tissues, and hematological parameters were not systematically performed. These assessments remain crucial for evaluating the long-term implications of UVB therapy and represent important considerations for future clinical translation and dose optimization protocols. Additionally, for clinical translation, several key aspects need to be addressed: (1) optimization of treatment parameters including radiation dose, frequency, and duration in human subjects; (2) establishment of standardized protocols for different patient populations; (3) evaluation of long-term safety and efficacy through well-designed clinical trials; and (4) development of personalized treatment strategies based on individual patient characteristics such as age, gender, and disease severity.

## 5 Conclusion

In conclusion, our study demonstrates that UVB irradiation, particularly with a 2-minute treatment protocol, effectively attenuates IVDD progression through potent activation of the NRF2/KEAP1 antioxidant pathway and subsequent suppression of NLRP3-mediated pyroptosis. These findings not only establish UVB irradiation as a promising therapeutic approach for IVDD treatment but also uncover its novel protective mechanism in degenerative disc disease. Future studies addressing the detailed molecular mechanisms, particularly the potential role of systemic vitamin D signaling, together with well-designed clinical trials will be essential for translating this therapeutic approach into clinical practice.

## Data Availability

The datasets presented in this study can be found in online repositories. The names of the repository/repositories and accession number(s) can be found in the article/supplementary material.
